# From In Vitro Cytotoxicity to In Vivo Zebrafish Assays: A Study on 3,3-Dichloro β-, γ- and δ-Lactams and Their Biological Activity Profiles

**DOI:** 10.3390/ph18040488

**Published:** 2025-03-28

**Authors:** Faïza Diaba, María del Carmen Morán, Elisabet Teixidó

**Affiliations:** 1 Laboratori de Química Orgànica, Facultat de Farmàcia i Ciències de l’Alimentació, IBUB, Universitat de Barcelona, Av. Joan XXIII 27-31, 08028 Barcelona, Spain; 2 Departament de Bioquímica i Fisiologia-Secció de Fisiologia, Facultat de Farmàcia i Ciències de l’Alimentaciò, Universitat de Barcelona, Avda. Joan XXIII 27-31, 08028 Barcelona, Spain; 3 Institut de Nanociència i Nanotecnologia—IN2UB, Universitat de Barcelona, Avda. Diagonal, 645, 08028 Barcelona, Spain; 4 GRET-Unitat de Toxicologia, Departamento de Farmacología, Toxicología y Química Terapéutica, Facultat de Farmàcia i Ciències de l’Alimentació, INSA, Universitat de Barcelona, Av. Joan XXIII 27-31, 08028 Barcelona, Spain; eteixido1511@ub.edu

**Keywords:** β-lactams, γ-lactams, δ-lactams, in vitro cytotoxicity, cancer, in vivo Zebrafish, hemocompatibility, selective toxicity

## Abstract

**Background/Objectives:** Anticancer research is a constantly evolving field due to cancer’s complexity and adaptability. This study aims to evaluate the hemolytic behavior and cytotoxic properties of ten 3,3-dichlorolactams against A431 tumor and 3T3 fibroblast cells, with a particular focus on their selective toxicity. **Methods:** To achieve this, we assessed the hemocompatibility and cytotoxic effects of the lactams, determining their impact on cell viability through MTT and NRU assays. Additionally, AO/EtBr double staining was used to confirm apoptosis as a mechanism of cell death. To complement the in vitro findings, in vivo experiments were conducted using Zebrafish embryos to evaluate acute, developmental, and neurotoxic effects. **Results:** The results demonstrated that all lactams were hemocompatible, with the cytotoxicity influenced mainly by their structure and the tested concentration. β-Lactam 1 was the most efficient in inducing selective toxicity against A431 cells, showing the lowest IC_50_ values (71 μg/mL and 210 μg/mL (MTT) and 35 μg/mL and >250 μg/mL (NRU) for A431 and 3T3 cell lines, respectively), with SI values close to 3 and >7. Moreover, cell death induction through apoptosis was confirmed by AO/EtBr double staining. Finally, despite its lower acute toxicity compared to other anticancer agents, the in vivo experiments revealed that **1** induced developmental toxicity and neurotoxic effects in Zebrafish embryos at concentrations lower than those affecting A431 cancer cells. **Conclusions:** the study highlights the potential of β-lactam derivatives as promising anticancer agents while emphasizing the need for comprehensive safety assessments. Future research should further explore structural modifications to enhance efficacy and specificity while minimizing adverse effects.

## 1. Introduction

**Figure 1:**
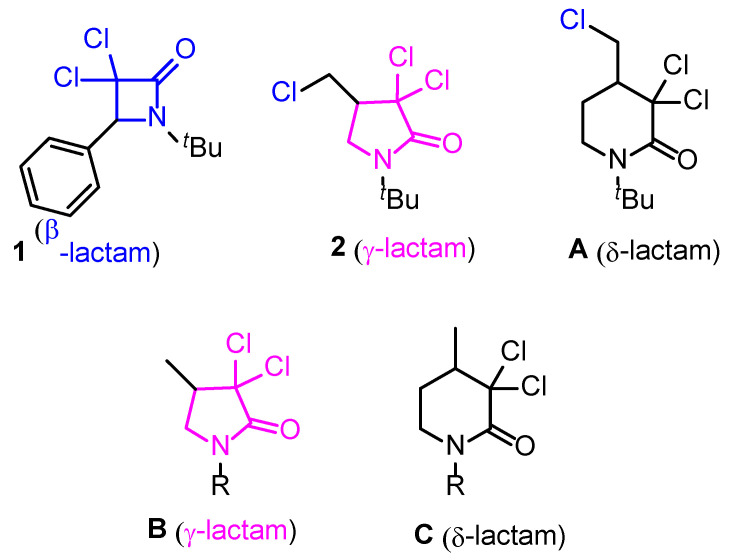
Previously investigated molecules (**1**, **2**, and **A**) and the general structures of the candidates involved in the present study (**B**,**C**).

Lactams, particularly β-lactams, are well known for their antibiotic properties but have also demonstrated their potential as anticancer agents. Recent studies have explored β-lactam derivatives for their therapeutic applications in cancer treatment, focusing on their mechanisms of action and structure–activity relationships [1]. Notably, certain β-lactam analogs have been shown to induce apoptosis in cancer cells by disrupting microtubule organization, leading to mitotic arrest [2]. Four years ago, we started an investigation into the synthesis and screening of a group of 3,3-dichloro-β-lactams regarding their cytotoxic activity against cancer cell lines [3]. After an evaluation of their effects on cell viability and their potential mechanisms of action using different endpoint methods, compound **1** (Figure 1) was found to be the most cytotoxic, showing the lowest IC_50_ values (20–49 g/mL and 30–47 g/mL) for the HaCaT and A431 cell lines, respectively. Thereafter, we extended our investigation to 4-(chloromethyl)-3,3-dichloro-γ-lactams prepared through atom transfer radical cyclization (ATRC) from tethered alkenyl trichloroacetamides in the presence of catalytic RuCl_2_(PPh_3_)_3_ and under microwave activation [4]. The investigation was undertaken on four 3,3-dichloro-γ-lactams, featuring different substituents on the nitrogen, along with δ-lactam **A**. Among the five products, γ-lactam **2** (Figure 1) exhibited the lowest IC_50_ values (100–250 µg/mL), with promising selectivity against squamous carcinoma cells (A431) in comparison with the fibroblast (3T3) cell line.

To further examine the impact of chlorine absence on γ- and δ-lactam activity, we expanded our study to assess the cytotoxic behavior of lactams type **B** and **C** (Figure 1), synthesized in our research group using photochemistry [5,6]. Their behavior was compared to that of their structural analogs, **2** and **A**, to gain deeper insights into structure-activity relationships. Notably, the presence or absence of a chlorine atom can significantly influence cytotoxic activity, with specific substitutions either enhancing or reducing potency [7]. For instance, in a study on imidazopyridine derivatives [8], the compound bearing a chlorine substituent was inactive against all tested cancer cell lines (IC_50_ > 100 µM), whereas its analog without this atom exhibited moderate cytotoxic activity against MCF-7 (40.3 µM) and HT-29 (61.2 µM).

## 2. Results and Discussion

**Figure 2:**
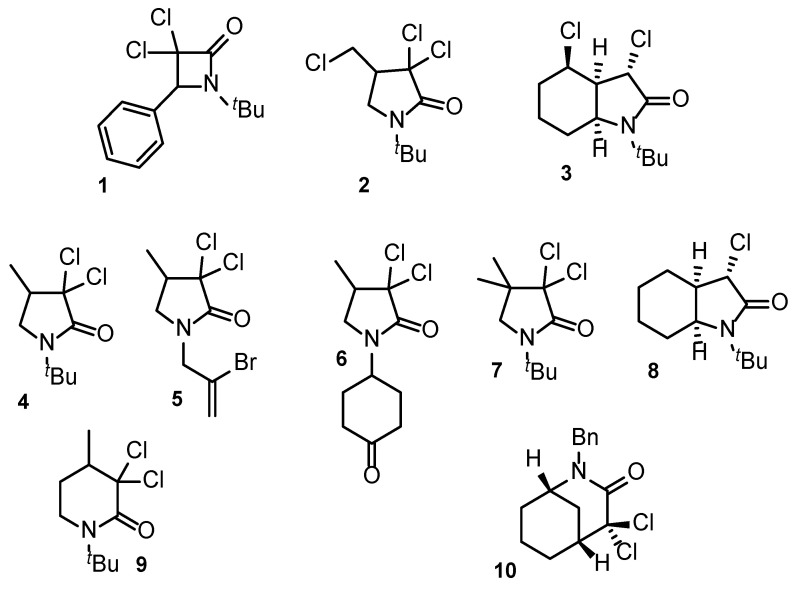
Selected β-, γ-, and δ-lactams used for hemocompatibility and cytotoxicity studies.

The study included lactams **1** and **2** and was expanded to lactam derivatives **3**–**10** (Figure 2) to analyze the structural diversity, such as nitrogen substitution, modifications on the 2-pyrrolidinone ring, the presence of an additional fused ring, and the size of the lactam heterocycle on their hemolytic and cytotoxic properties.

### 2.1. Hemocompatibility Studies

As previously noted, lactam compounds, especially β-lactams, play a vital role in modern medicine as essential components in antibiotics such as penicillins and cephalosporins. However, their therapeutic potential can be hindered by toxicity concerns, including hemolytic activity, which can damage red blood cells. Certain β-lactams have been associated with hemolytic reactions, although such occurrences are relatively rare. For instance, ceftaroline, a fifth-generation cephalosporin, has been associated with positive direct Coombs test results, suggesting potential immune-mediated hemolytic anemia [9]. Similarly, cefotetan, a second-generation cephalosporin antibiotic of the cephamycin subclass, has also been reported to cause this condition [10]. Moreover, drug-induced immune hemolytic anemia (DIIHA) was reported with other β-lactam antibiotics, including amoxicillin-clavulanate and ceftazidime [11].

**Figure 3:**
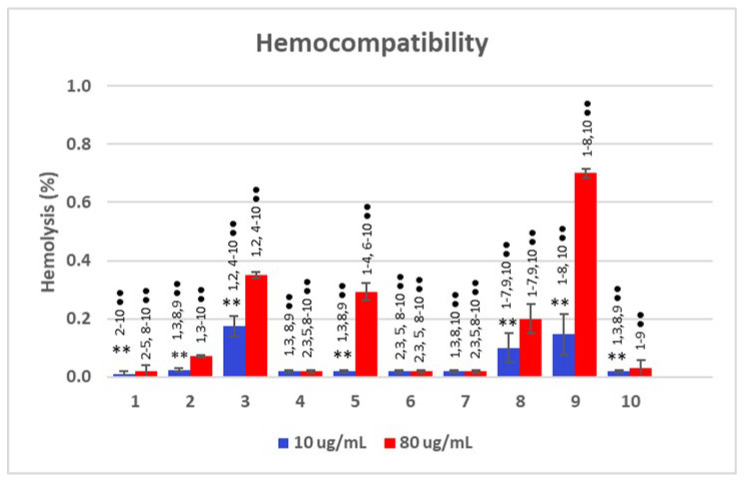
The percentage of hemolysis induced by compounds **1**–**10** as a function of the concentration. The data correspond to the average of three independent experiments ± standard deviation. ** (*p* < 0.001) indicates significant differences between concentrations for the same compound. ●● (*p* < 0.001) indicates significant differences between compounds for the same concentration.

In this context, non-hemolytic compounds are essential, as they enhance the safety and efficacy of lactam-based drugs by reducing adverse effects while maintaining their antitumor activity. In accordance with EU regulations (ISO 10993-4) [12], the assessment of the biological compatibility of new compounds that may come into contact with the blood necessitates in vitro hemocompatibility evaluations. In this study, the extent of hemolysis induced by the different compounds was assessed by incubating them with an erythrocyte suspension. The hemolytic response of compounds **1**–**10** was evaluated at two concentrations (10 and 80 µg/mL), the results of which are presented in Figure 3.

These results indicate that most compounds exhibited minimal hemolysis, suggesting good hemocompatibility. However, there are some notable differences between the two tested concentrations. In general, the hemolysis percentage is higher at 80 µg/mL compared to 10 µg/mL, indicating a concentration-dependent effect. The degree of hemolysis fluctuated moderately, with values ranging between 0.01% (compound **1**), 0.02% (compounds **2**, **4**–**7**, and **10**), and 0.1% (compounds **3**, **8**, and **9**) at concentrations equal to 10 µg/mL. At this dose, compounds **1**, **3**, **8**, and **9** demonstrated significant differences (*p* < 0.001) with regard to the other compounds. As the concentration increased to 80 µg/mL, the hemolytic response remained stable for compounds **1**, **4**, **6**, **7**, and **10**, while it doubled for compounds **3** and **8** and increased three- and fivefold for compounds **2** and **9**, respectively.

Specifically, compounds **3**, **5**, and **9** showed the highest levels of hemolysis, with sample **9** at 80 µg/mL reaching nearly 0.7%, which is significantly higher than all the other samples. Compounds **3**, **5**, **8**, and **9** exhibited significant differences (*p* < 0.001) from the rest of the compounds. When comparing the effects of varying concentrations, significant differences (*p* < 0.001) between the hemolytic responses at the two different concentrations were found for all the compounds, except for compounds **4**, **6**, and **7**.

The hemolytic response seems to be influenced by factors such as the size of the lactam ring, the nature of the nitrogen substituent, and the presence of a third chlorine atom. However, the results indicate that the proposed compounds exhibit non-hemolytic properties. This conclusion is supported by the classification criteria for non-hemolytic compounds, which generally require hemolysis values to remain below 2% [13]. In addition, the value of HC_50_, which refers to the concentration of a substance that causes hemolysis in 50% of red blood cells, could be defined as higher than 80 µg/mL in all cases.

### 2.2. Cell Viability Assays

Lactams could play a significant role in medicinal chemistry, particularly in the development of antibiotics and anticancer agents. Cytotoxicity studies of lactams are essential to evaluate their safety and potential therapeutic effects. These studies assess how lactams affect cell viability, proliferation, and apoptosis in various biological systems. Cytotoxicity studies help determine the safety and therapeutic window of these compounds, ensuring that they selectively target pathogens or cancer cells without harming healthy cells [3,4].

In view of the potential antitumor activity of the proposed compounds, commercially available cell lines with tumor characteristics and normal characteristics were chosen. This work includes keratinocytes with tumor characteristics resembling those found in squamous cell carcinoma (SCC), namely, the A431 cell line. SCC stands out as the most prevalent form of skin cancer, surpassing all other types in frequency [14]. For comparative purposes, and to assess potential selective toxicity, the murine Swiss albino fibroblast (3T3) cell line was also included in these screening assays. 3T3 fibroblasts are readily available and are closely representative of a physiological model cell line [15].

Two different endpoints, 3-(4,5-dimethyl-2-thiazolyl)-2,5-diphenyltetrazolium bromide (MTT) and neutral red uptake (NRU), were used to assess differences in cell-induced cytotoxicity. MTT reveals details about the modification of the metabolic activity of the mitochondria inside the cells upon incubation with drugs. In addition, NRU evaluates the interaction of these drugs with the plasmatic membrane. Cell responses were determined from the MTT [16] and NRU [17] assays, using the two proposed cell lines. Cytotoxicity assays were performed at concentrations equal to 80 and 250 µg/mL. The IC_50_ value, referring to the half-maximal inhibitory concentration, was determined as a key parameter in cytotoxicity studies (Table 1). It plays a crucial role in evaluating cell viability and assessing cellular responses to various drugs or toxins. For cases where IC_50_ values were below 80 µg/mL, additional studies at lower concentrations were conducted to accurately determine these values. Conversely, when IC_50_ values exceeded 250 µg/mL and could not be precisely defined, they were considered higher than the highest tested concentration (250 µg/mL), as shown in Table 1. The selectivity index (SI), used as a measurement of action against the tumor cell line in comparison with a normal cell line, was also included.

When the effect of the endpoint method on IC_50_ values was compared, both the MTT and NRU assays followed similar trends. However, the lack of information on some of the NRU data (always above 250 µg/mL), demonstrated poor interaction with the plasma membrane and lysosomal accumulation (NRU method) compared to the modification of the metabolic activity of the mitochondria inside the cells (MTT method). These results were assessed in light of those found previously in our laboratory concerning β-lactam derivatives [3], for which no differences in the mode of action against the plasmatic membrane (NRU) and metabolic response (MTT) were determined. Compounds **4**, **5**, **6**, and **8** exhibited weak or no cytotoxicity, as their IC_50_ values were always above 250 µg/mL. However, compounds **1**, **7**, **9**, and **10** showed lower IC_50_ values for A431 in comparison with 3T3 cells, demonstrating some selective cytotoxicity.

This selective behavior could be highlighted by considering the selectivity index (SI) (Table 1). Based on the ratio between the IC_50_ values of both normal and tumor cell lines, the most promising compounds can be selected by considering SI values higher than 1. Compounds **8**, **9**, and **10** demonstrated mild selectivity against tumor cells with SI values slightly above 1 using the MTT method. However, compound **1**, with SI values close to 3.0 (MTT) or almost 7 (NRU) exhibited promising selectivity in its mode of action through A431 cells, in comparison with 3T3 cells. In contrast, compound **3**, which showed SI values lower than 1 (MTT method), indicated a lack of selectivity in its mechanism of action.

**Figure 4:**
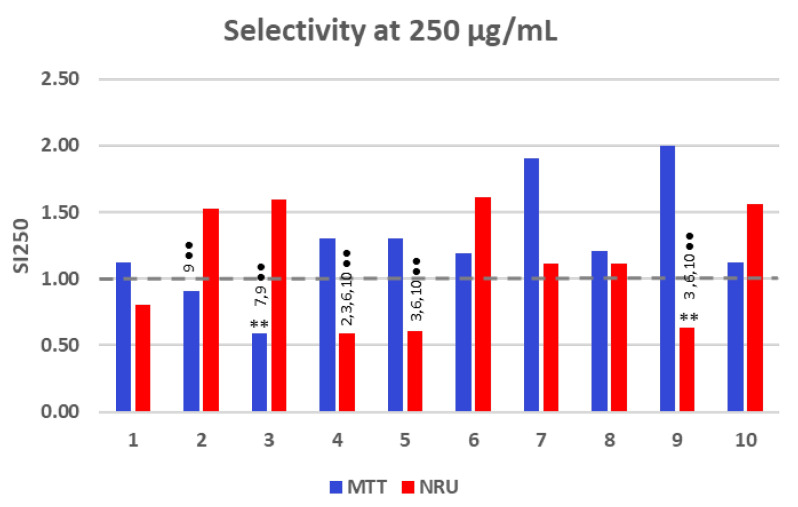
Selective toxicity at a concentration of 250 µg/mL against the A431 cell line compared to the 3T3 fibroblast cell line when incubated with compounds **1**–**10** for 24 h, as determined by MTT and NRU assays. ** (*p* < 0.001) indicates significant differences between methods for the same compound. ●● (*p* < 0.001) indicates significant differences between compounds for the same method.

Since a defined SI value could not be determined in several cases due to the lack of IC_50_ values, a pseudo-selectivity index (SI) at the highest tested concentration (SI_250_) was calculated for all compounds. Figure 4 presents the selectivity values for compounds **1**–**10**, as determined by the MTT and NRU methods.

The obtained results suggested that the selectivity at the highest assayed concentration (250 µg/mL) varied across compounds **1**–**10**. As a general trend, the MTT endpoint method provided SI_250_ values higher than 1 in almost all cases, whereas the NRU values appeared to be more consistently below or around 1, implying either lower selectivity or a different sensitivity in the assay. Considering differences in the mode of action between methods, the metabolic activity (MTT) assay provides greater selectivity than the membrane integrity-based NRU assay, suggesting a potential discrepancy in the cytotoxicity mechanisms. The highest SI values (above 1.5) in the MTT assay suggest strong selectivity under some conditions, particularly for lactams **7** and **9**. The NRU assay shows less fluctuation than the MTT method but still exhibits notable differences across conditions. Compounds **2**, **3**, **6**, and **10** exhibited the highest SI_250_ values using this method.

When the effect of the endpoint method was compared, the statistical analyses demonstrated significant differences (*p* < 0.001) between the methods for compounds **2**, **3**, and **9**, with SI_250_ values that were higher for NRU (compounds **2** and **3**) and MTT (compound **9)**. These findings suggest that the choice of cytotoxicity assay can influence the interpretation of results and underscore the importance of selecting appropriate methods depending on the chemical profile of the lactam. Using the NRU method, significant differences (*p* < 0.001) were observed for compounds **4**, **5**, and **9** compared to compounds **3**, **6**, and **10**. These results suggest that the cyclohexanone substituent in compound **6** is better than the *t*-Butyl group in compound **4** or the bromoallyl chain in compound **5**. Significant differences were also observed between compound **4** and compound **2**. The presence of the third chlorine atom seems to improve the selectivity. Using the MTT method, significant differences (*p* < 0.001) were observed between compounds **2** and **9**, as well as between compound **3** and compounds **7** and **9**. The molecular profile or structural variations may also influence the MTT results, as differences in functional groups, electronic properties, or steric hindrance can affect cellular metabolism, mitochondrial activity, or interactions with the assay reagents.

With regard to the structure–activity relationship, the results obtained in this study lead us to the following conclusion: the *t*-butyl group remains the most effective nitrogen substituent, as demonstrated by the optimal results obtained from compounds **1**, **7**, and **9** with the MTT method (Table 1). As for the lactam ring size, our results demonstrate that selectivity decreases as the ring expands from β-lactam to δ-lactam, transitioning through γ-lactam. This trend is particularly evident in the results for compounds **1**, **4**, and **9**. Moreover, the presence of an additional methyl group in the lactam ring seems to enhance SI, as seen in compound **7** in comparison with compound **4**. With respect to the additional six-membered fused ring, as seen in derivatives **8** and **10**, this appears to enhance selectivity. Finally, using the MTT method, it is clear that the presence of the third chlorine atom does not enhance the cytotoxic activity, as demonstrated in compound **2** in comparison with lactam **4**. The same trend is observed for lactams **A** and **3** in comparison with compounds **9** and **8**, respectively.

### 2.3. Cellular Death Events Induced by Lactam **1**

When cells are exposed to cytotoxic agents, there are various patterns of cell death that they may undergo. The two major types of cell death are apoptosis and necrosis. Necrosis is usually followed by an inflammatory response to the released cellular contents, often resulting in further tissue damage [18,19]. Tumors with a high incidence of necrosis rather than apoptosis show aggressive behavior and generally have a poor prognosis [20]. Thus, the ability to distinguish between these two modes of cell death, especially at very early time points post-treatment, may provide useful information on the success of cancer treatment.

**Figure 5:**
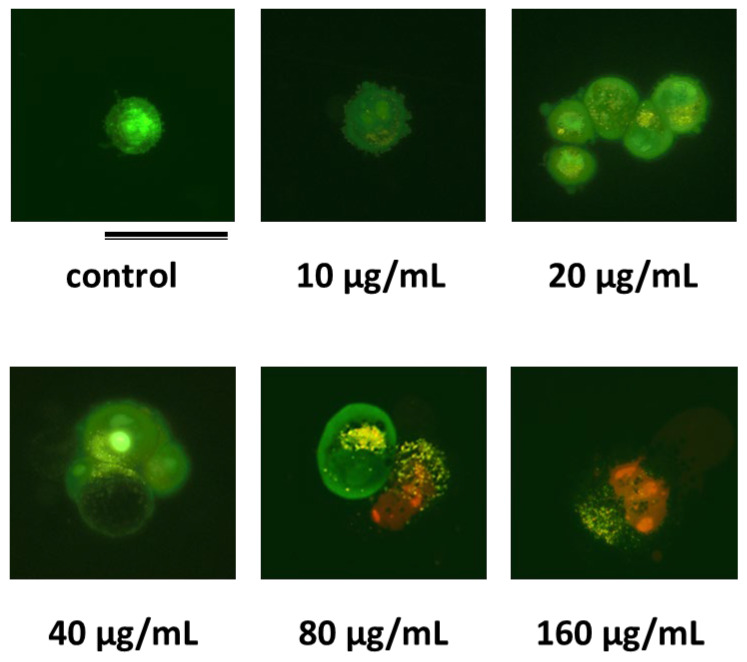
Representative fluorescence microscopy images of unexposed (control) and incubated A431 cells in the presence of compound **1** at different concentrations, using acridine orange/ethidium bromide (AO/EB) double staining. The bar scale corresponds to 25 µm.

Considering the obtained results concerning the selective behavior of compound **1** (Table 1), tumor cells were incubated in the presence of compound **1** at different concentrations below and above their IC_50_ value, and were then stained with the nucleic acid-binding fluorochromes AO and EtBr to evaluate the induced morphological alterations that could be attributed to one mechanism or another [21,22]. Figure 5 shows representative fluorescence microscopy images under the assayed conditions. The untreated cells mainly exhibited a green fluorescence, due to the exclusion of EB but not of AO. Viable cells showed a light green nucleus with an intact structure and presented punctate orange-red fluorescence in the cytoplasm, representing lysosomes stained by AO. Following treatment with compound **1,** an increase in the number of apoptotic cells has been revealed, showing early apoptotic live cells (green) with chromatin super-aggregation, i.e., highly condensed chromatin at the lowest compound **1** concentration. By increasing the concentration (20–40 µg/mL), a number of early apoptotic cells with fragmented nuclei exhibited punctate or bubbly AO distribution in the cytosol. Apoptotic cells with a split nucleus and weak AO staining also exhibited punctate or aggregated AO staining in the cytosol. Above the IC_50_ values (80 μg/mL), red fluorescence begins to appear, likely indicating compromised membrane integrity, revealed through propidium iodide staining. This is associated with the transition from apoptosis to secondary necrosis. At the highest assayed concentration (160 µg/mL), bright red fluorescence dominated, pointing to necrotic cell death with a significant loss of membrane integrity.

The observed results strongly suggest that compound **1** induces cell death primarily via apoptosis, as evidenced by chromatin condensation, nuclear fragmentation, and the stepwise loss of membrane integrity with increasing concentration. Necrotic features were minimal and likely secondary, occurring at later stages of apoptosis [23].

### 2.4. Zebrafish Experiments

Zebrafish provide a widely used vertebrate model for cost-effective, in vivo whole-organism screening. Using the fish embryo acute toxicity (FET) assay, following the modified OECD Test Guideline 236 [24], the acute toxicity of compound **1** was evaluated. The lethality and dysmorphogenesis of this compound were assessed by exposing fertilized Zebrafish embryos to increasing concentrations of the compound from 1.7 to 27.2 µg/mL, from 2 to 72 h post-fertilization (hpf).

**Figure 6:**
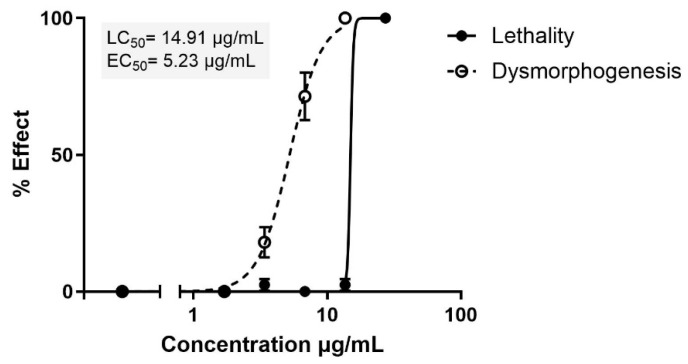
Concentration-response curves for lethality and dysmorphogenesis upon exposure of Zebrafish embryos to β-lactam **1** in the range of 1.7–27.2 µg/mL. The results are shown as the mean ± SEM of at least three independent experiments, using 10 embryos for each concentration in each experiment. LC_50_ and EC_50_ were calculated by adjusting the results of the means of the tested groups to a non-linear concentration-response curve with variable slope and least-squares fit, fixing the minimum and maximum at 0 and 100% lethality.

At 72 hpf, a concentration-response relationship was observed for lethality and dysmorphogenesis, with an LC_50_ (lethal concentration, the concentration killing 50% of individuals) of 14.91 µg/mL, and EC_50_ (effective concentration causing dysmorphogenesis in 50% of larvae) of 5.23 µg/mL, respectively (Figure 6). This resulted in a teratogenic index (TI) of 2.85, leading to a classification of potential teratogen (TI > 2) [25].

**Figure 7:**
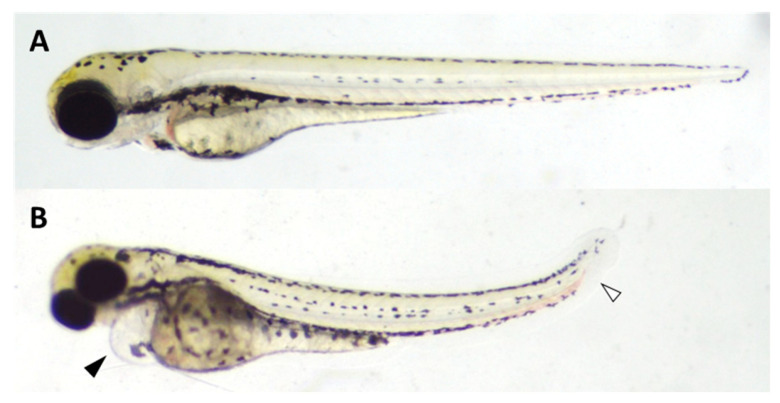
Representative morphological effects observed in Zebrafish embryos exposed to solvent control (1% DMSO) (**A**) and 13.6 μg/mL of 3,3-dichloro β-lactam **1** (**B**) from 2 to 72 hpf. The black arrow indicates pericardial edema and the white arrow indicates flexion abnormality.

Morphological abnormalities primarily included pericardial edema, large yolk sacs, and flexion abnormalities (Figure 7). The compound showed low acute toxicity when compared to other anticancer agents such as taxol (1.05 µg/mL) [26] or doxorubicin (5.6 µg/mL) [27]. The LC_50_ in Zebrafish embryos (14.9 µg/mL) was lower than the in vitro cytotoxic concentrations in A431 cells (71.65 µg/mL using the MTT method and 35.74 µg/mL using the NRU method), while the EC_50_ for developmental effects was even lower (5.23 µg/mL). Zebrafish embryogenesis follows a well-defined developmental sequence guided by various signaling pathways, making it a valuable model for studying developmental toxicity. Unlike single-cell cytotoxicity assays, Zebrafish provide a whole-organism context with multiple cell types, allowing the differentiation of toxicity effects across various developing tissues. The disruption or alteration of these pathways due to their exposure to potentially toxic agents, such as an anticancer compound, can result in specific developmental defects in the embryos [28].

**Figure 8:**
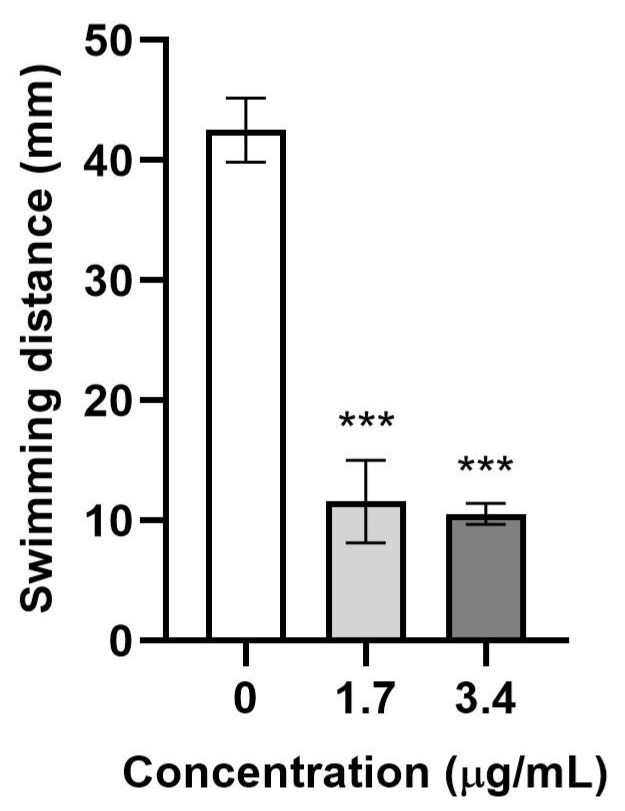
Evaluation of the TER after exposure to β-lactam **1**. Results are presented as the mean ± SEM of swimming distance in millimeters. *** *p* < 0.001. The experiment was conducted three times with six embryos per concentration group, with each embryo receiving three tactile stimuli.

Additionally, at 74 hpf, following a 2-h washout period, the response to a tactile stimulus or touch-evoked response, also known as a TER, was evaluated in embryos. This assay requires the embryos to have proper neuromuscular function. A specific significant neurodevelopmental adverse effect was observed in the TER at all tested concentrations up to 3.4 µg/mL, following the criteria described in Section 3, Materials and Methods (Figure 8). Further studies might be needed to elucidate the specific mechanisms by which the compound affects neurodevelopment or motor function and to determine whether these effects translate to other organisms. These findings are consistent with the broader body of research on anticancer agents [29], highlighting the importance of neurotoxicity evaluation in drug development and reinforcing the need for ongoing research to address these concerns. The videos and figures related to this part of the work are available in the Supplementary Materials.

The advantage of using the Zebrafish model in this study lies in its ability to simultaneously evaluate developmental and neurotoxic effects in a whole-organism context, providing a comprehensive assessment of the compound’s potential risks.

## 3. Materials and Methods

### 3.1. Materials and Methods Used for the Biological Assays

#### 3.1.1. Materials

Dulbecco’s modified Eagle’s medium (DMEM), fetal bovine serum (FBS), L-glutamine solution (200 mM), trypsin–EDTA solution (170,000 U/L trypsin and 0.2 g/L EDTA), penicillin–streptomycin solution (10,000 U/mL penicillin and 10 mg/mL streptomycin), and phosphate-buffered saline (PBS) were obtained from Lonza (Verviers, Belgium). 3-(4,5-Dimethyl-2-thiazolyl)-2,5-diphenyltetrazolium bromide (MTT) and neutral red dye (NR) were obtained from Sigma–Aldrich (St. Louis, MO, USA). The 75 cm^2^ flasks and 24- and 96-well plates were obtained from TPP (Trasadingen, Switzerland). All other reagents were of analytical grade.

#### 3.1.2. Methods

• In Vitro *Assay Using Human Erythrocytes*
- Acquisition and Extraction of the Erythrocytes
  Human blood samples were obtained from the Banc de Sang i Teixits de Barcelona (Spain) from the Catalan Department of Health. Blood was deposited in tubes with the anticoagulant EDTA-K3. Blood samples were centrifuged at 3000 rpm and 4 °C for 10 min (Megafuge 2.0 R. Heraeus Instruments, Hanau, Germany) to induce sedimentation. Plasma was extracted with a Pasteur pipette. Next, the residual pellet was washed with PBS at pH 7.4. This procedure was repeated three times to remove residual leukocytes and platelets and to concentrate the erythrocytes. Following the last wash, the erythrocyte suspension was diluted (1:1) in PBS at pH 7.4 to obtain a suitable erythrocyte suspension (cell density of 8 × 10^9^ cell/mL).
- Hemolysis Assay
  The hemolysis assay determined the capability of different compounds to induce the hemolysis of the erythrocyte membrane. Stock solutions of each compound at 1 mg/mL in PBS at pH 7.4 were prepared. Different volumes (10–80 μL) were placed in polystyrene tubes, and an aliquot of 25 µL of the erythrocyte suspensions was added to each tube. The final volume was 1 mL. The tubes were incubated at room temperature under rotatory conditions. Then, the tubes were centrifuged at 10,000 rpm for 5 min. The supernatants’ absorbance at 540 nm (Shimadzu UV-160A, Shimadzu, Duisburg, Germany) was compared with that of the positive (erythrocytes hemolyzed with distilled water) and negative (erythrocyte suspension in PBS at pH 7.4) controls.
  The degree of hemolysis was determined using the following equation:
  Hemolysis (%) = 100 × (Abs − Abs_0_)/(Abs_100_ − Abs_0_)
  where Abs, Abs_0_, and Abs_100_ are the absorbance of the test samples, of the suspension treated with isotonic phosphate–buffered saline (PBS), and of the suspension of complete hemolysis treated with distilled water, respectively.
• In Vitro *Essay Using Cell Cultures*
  The murine Swiss albino fibroblasts (3T3) and the squamous cell carcinoma (A431) cell lines were obtained from Celltec UB. Cells were grown in DMEM medium (4.5 g/L glucose) supplemented with 10% (v/v) FBS, 2 mM L-glutamine, 100 U/mL penicillin, and 100 µg/mL streptomycin at 37 °C with 5% CO_2_. Cells were routinely cultured in 75 cm^2^ culture flasks and were trypsinized using trypsin–EDTA when the cells reached approximately 80% confluence. The trypan blue assay, which allows for the direct identification and enumeration of the live (unstained) and dead (blue) cells in the cell population, was used to evaluate the viability of the cells in the cell suspension obtained.
- Cell Viability Assays
  Cell lines of 3T3 (1 × 10^5^ cells/mL) and A431 (5 × 10^4^ cells/mL) were grown at defined densities in the 60 central wells of a 96-well plate. The cells were incubated for 24 h in 5% CO_2_ at 37 °C. Then, the spent medium was removed, and the cells were incubated for 24 h, with their corresponding compound solutions (1 mg/mL) previously diluted in a minimum amount of DMF (dimethylformamide) and then in DMEM medium supplemented with 5% FBS (100 µL) at the required concentration range (80 or 250 µg/mL). The viability of the cells upon incubation with the lactam derivatives was assayed using 2 different endpoints: NRU and MTT.
• *NRU Assay*
  The neutral red uptake (NRU) assay is based on the accumulation of dye in the lysosomes of viable cells. After the cells were incubated for 24 h with the corresponding systems, the medium was removed and the solutions were incubated with the NR dye (Sigma-Aldrich, St. Louis, MO, USA) solution (50 µg/mL) dissolved in the medium, without FBS and without phenol red (Lonza, Verviers, Belgium), for 3 h. The cells were then washed with sterile PBS, followed by the addition of 100 µL of a solution containing 50% absolute ethanol and 1% acetic acid in distilled water to extract the dye. To promote the total dissolution of the dye, the plates were placed in a microtiter plate shaker for 5 min at room temperature. The absorbance of the resulting solutions was measured at 550 nm (Bio-Rad 550 microplate reader, Bio-Rad California, Hercules, CA, USA). Finally, the effect of each treatment was calculated as the percentage of dye uptake by viable cells relative to the control cells (cells without any treatment).
• *MTT Assay*
  Only living cells can reduce the yellow tetrazolium salt 3-(4,5-dimethyl-2-thiazolyl)-2,5-diphenyltetrazolium bromide (MTT) to insoluble purple formazan crystals. After a 24-h incubation of the cells with their corresponding NPs, the medium was removed and 100 µL of MTT (Sigma-Aldrich, St. Louis, USA) in PBS (5 mg/mL), diluted 1:10 in culture medium without phenol red and without FBS (Lonza, Verviers, Belgium), was added to the cells. After 3 h of incubation, the medium was removed. Thereafter, 100 µL of DMSO (Sigma-Aldrich, St. Louis, MO, USA) was added to each well to dissolve the purple formazan crystals. Agitation and determination of the absorbance of the extracted solution were performed under the same conditions, as described in the NRU assay section above. Finally, the effect of each treatment was calculated as the percentage of reduction of tetrazolium salt by viable cells relative to control cells (cells without any treatment).
- Selectivity towards Cancer Cells
  The corresponding half-maximal inhibitory concentration (IC_50_) values for the different formulations as a function of the cell line and endpoint were determined from the fitting of concentration-dependent viability curves. The corresponding selectivity indexes toward tumor cells were calculated using the following ratio:
  SI = IC_50_ (normal cell line)/IC_50_ (tumor cell line)
  where 3T3 fibroblasts were used as closely representative cells under non-tumor conditions. When the corresponding IC_50_ values were not able to be determined, pseudo selectivity indexes toward tumor cells were calculated, considering the discrete cellular responses at the maximum tested concentration (250 µg/mL).
- Cellular death events induced by the selected lactam **1**
  Changes in the morphology of tumor cells induced by selected lactams were evaluated using the acridine orange/ethidium bromide (AO/EB) double staining, according to standard procedure, and using fluorescence microscopy. Cells were seeded at the densities mentioned above in 24-well plates, following the standard atmosphere, temperature, and time conditions. Then, the cells were incubated with the selected lactam in DMEM medium supplemented with 5% FBS at the required concentration range (0, 10, 20, 40, and 80 µg/mL). After 24 h of incubation, the cells were trypsinized. Then, the fluorescent dyes AO (0.5 μg/mL) and BE (10 μg/mL) were added to the cellular suspension. The freshly stained cell suspension was dropped on a glass slide and covered by a cover slip. Slides were observed with an Olympus BX41 microscope equipped with a UV-mercury lamp (100 W Ushio Olympus, Olympus Iberia, Barcelona, Spain) and a U-N51004v2-FlTC/TRITC-type filter set (FITC: BP480-495, DM500-545, BA515-535, and TRITC: BP550-570, DM575-, BA590-621). Images were digitized on a computer through a video camera (Olympus digital camera XC50, Olympus Iberia, Barcelona, Spain) and were analyzed with an image processor (Cell-B analysis).
• *Statistical Analysis*
  Experiments were performed three times on independent occasions unless otherwise stated. The results are expressed as means ± standard deviation. A one-way analysis of variance (ANOVA) was used to determine the statistical differences between data sets, followed by Scheffé post hoc tests for multiple comparisons. IBM SPSS Statistics software version 29.0 (New York, NY, USA) was used to perform statistical analysis. Differences were considered statistically significant at *p* < 0.001. Significant differences are illustrated in the figures using an asterisk or other superscript symbols.

### 3.2. Materials and Methods for In Vivo Essays

- *Zebrafish egg production and exposure*
  The study was conducted in accordance with the local legislation and institutional requirements, according to the Generalitat de Catalunya Decree 53/2013, which regulates the use of animals for experimental and other scientific purposes.
  Adult Zebrafish, both male and female, were procured from a commercial supplier (Pisciber, Barcelona) and housed in a closed flow-through system, with standardized dilution water as specified in ISO 7346-1 [30] (2 mM CaCl_2_•2 H_2_O; 0.5 mM MgSO_4_•7 H_2_O; 0.75 mM NaHCO_3_; 0.07 mM KCl). The fish were maintained at a temperature of 26 ± 1 °C on a 14-h light and 10-h dark cycle and were fed daily in the morning with Artemia salina, then with commercial flake food in the afternoon (SDS400, Special Diet Services, Dietex, France). For optimal Zebrafish reproduction, we used animals between 6 months and a year old. The day before the eggs were needed, males and females were placed in a breeding tank with plants at a 2:1 male-to-female ratio (usually using 8 males and 4 females in a big breeding tank). The following morning, eggs were collected 30 min after the lights were turned on. The eggs were then cleaned successively with reconstituted water according to ISO standard 7346-1. Fertilized, non-coagulated, and synchronously divided eggs were selected using a stereomicroscope (Motic SMZ168, Motic Hong Kong Ltd., Hong Kong, China) and transferred to 6-well plates (10 embryos/well). The embryos were exposed to freshly prepared tested concentrations of the compound in 0.3× Danieau’s solution. The chemical stock solution was prepared in 100% dimethylsulfoxide (DMSO) and subsequently diluted in Danieau’s buffer with a final DMSO concentration of 1% (v/v). Testing was carried out using five concentrations with a negative control of solvent with 1% of DMSO. Embryos were incubated at 27 ± 1 °C with a light:dark cycle of 14:10 h until 72 h post-fertilization (hpf) under semi-static conditions, as exposure solutions were renewed with freshly prepared solutions every 24 h.
- *Lethality and dysmorphogenesis evaluation*
  At 24, 48, and 72 hpf, the embryos were observed under a stereomicroscope to assess lethality. The lethality criteria, based on OECD Test Guideline No. 236 [24], included coagulation of embryos, the absence of somite formation, and non-detachment of the tail starting from 24 hpf, with the addition of a lack of heartbeat from 48 hpf onward [29]. At the end of the test, dysmorphogenesis was evaluated in the live embryos only. The average lethality and dysmorphogenesis were calculated for 10 embryos per concentration, followed by averaging the results of at least three independent experiments. A sigmoidal (variable slope) curve fit was applied to these means to generate concentration-response curves and to calculate the LC_50_ (the concentration that causes lethality in 50% of the larvae) and EC_50_ (the concentration causing dysmorphogenesis in 50% of the larvae). The teratogenic index (TI) was determined as the ratio between the LC_50_ and EC_50_ (TI = LC_50_/EC_50_).
- *Touch-Evoked Response (TER) test*
  At 72 hpf, the test solutions were replaced with 0.3× Danieau’s solution without the chemical and incubated at 27 °C ± 1 °C for 2 h. Subsequently, the TER test was conducted according to the protocol described by Guzman et al. [31]. In brief, videos were recorded using a Casio Exilim EX-ZR200 video camera, and the distance swum by Zebrafish embryos was measured after three mechanical stimuli were applied to the tail with a forceps tip (Fine Science Tools, Dumont #5) at 10-s intervals. The distance was averaged and converted from pixels to millimeters. This assay was performed only on larvae from concentration groups where both the lethality and dysmorphogenesis were less than 20%. Six larvae per concentration group were evaluated in at least three independent experiments.
- *Statistical analysis*
  Statistical analysis was performed using GraphPad Prism v.10.0.2. The TER test was analyzed using a one-way ANOVA and a post hoc multiple-comparison Dunnett test. The significance threshold was established at *p* < 0.05.

## 4. Conclusions

In this work, a series of 3,3-dichloro β-, γ-, and δ-lactams were evaluated for their hemolytic activity and cytotoxic properties under both in vitro and in vivo conditions. This analysis highlights key structural factors influencing the selectivity index (SI) against A431 tumor cells, including nitrogen-ring substitution, ring size, functional groups, and overall molecular complexity. The results showed that compounds **1–10** have non-hemolytic properties and that the cytotoxic response is highly dependent on the structure and concentration of these lactams. Compound **1** remains the most effective in inducing selective toxicity against A431 cells, exhibiting the lowest IC_50_ values in both the MTT and NRU assays for the A431 and 3T3 cell lines, respectively, with a selectivity index significantly favoring tumor cells. Morphological studies based on AO/EtBr staining on A431 cells by incubation with compound **1** strongly suggest that the latter induces cell death primarily via apoptosis. Furthermore, the in vivo experiments conducted with Zebrafish embryos demonstrated that β-lactam **1** exhibited lower acute toxicity compared to other anticancer agents such as taxol or doxorubicin. However, lactam **1** showed developmental toxicity and developmental effects at concentrations lower than those affecting human A431 cancer cells. These findings indicate that developing organisms are more vulnerable than cancer cells to this compound and there is a need for additional research to determine its safety profile and potential mechanisms of toxicity. Future research will focus on expanding the SAR analysis by synthesizing and evaluating additional analogs of β-lactam **1** to refine the structure–activity relationships. Furthermore, computational docking studies will be conducted to better understand the binding interactions of these compounds with key molecular targets in cancer cells, providing insights into their potential mechanisms of action and selectivity.

## Figures and Tables

**Table 1:** Cell viability assessment: half-maximal inhibitory concentration (IC_50_) values of the corresponding lactam derivatives as a function of their cell line and endpoint method. The selectivity index (SI) against the tumor cell line, in comparison with a normal cell line (3T3), was also included.

Lactam	IC_50 (μg/mL)_ (MTT)		IC_50 (μg/mL)_ (NRU)		SI (MTT)	SI (NRU)
	A431	3T3	A431	3T3		
**1**	71.65 ± 1.23	210.42 ± 2.40	35.74 ± 3.24	>>250	2.94	>>6.99
**2**	>250	>250	231.66 ± 4.23	>250	n.d. ^1^	>1.08
**3**	250 ± 2.11	183.45 ± 3.11	>>250	>>250	0.73	n.d.
**4**	>250	>>250	>>250	>250	n.d.	n.d.
**5**	250 ± 1.32	>250	>>250	250	n.d.	n.d.
**6**	>250	>250	>>250	>>250	n.d.	n.d.
**7**	230.30 ± 2.54	>250	>>250	>>250	>1.08	n.d.
**8**	250 ± 1.78	>250	>>250	>>250	>1.0	n.d.
**9**	242.93 ± 1.78	>250	>>250	>250	>1.029	n.d.
**10**	238.29 ± 1.56	>250	>>250	>>250	>1.05	n.d.

^1^ n.d.: not determined.

## Data Availability

Data are contained within the article and the Supplementary Materials.

## Supplementary Materials

The following supporting information can be downloaded at: https://www.mdpi.com/article/10.3390/ph18040488/s1, Video CIMG6637.MOV: Touch-evoked response of control Zebrafish larvae; Video CIMG6649.MOV: Touch-evoked response of Zebrafish larvae exposed to 3.4 μg/mL of β-lactam **1** for 72 hpf; Figure CNT_2_1: Example of TER analysis in control larvae showing the traveled distance; Figure CONC12.5_1_3: Example of TER analysis in larvae exposed to 3.4 μg/mL of β-lactam **1**.
