# Supplementary figures and images for: From In Vitro Cytotoxicity to In Vivo Zebrafish Assays: A Study on 3,3-Dichloro β-, γ- and δ-Lactams and Their Biological Activity Profiles
Source: Pharmaceuticals (Basel). 2025 Mar 28;18(4):488. doi: 10.3390/ph18040488 (PMC12030062; doi:10.3390/ph18040488)

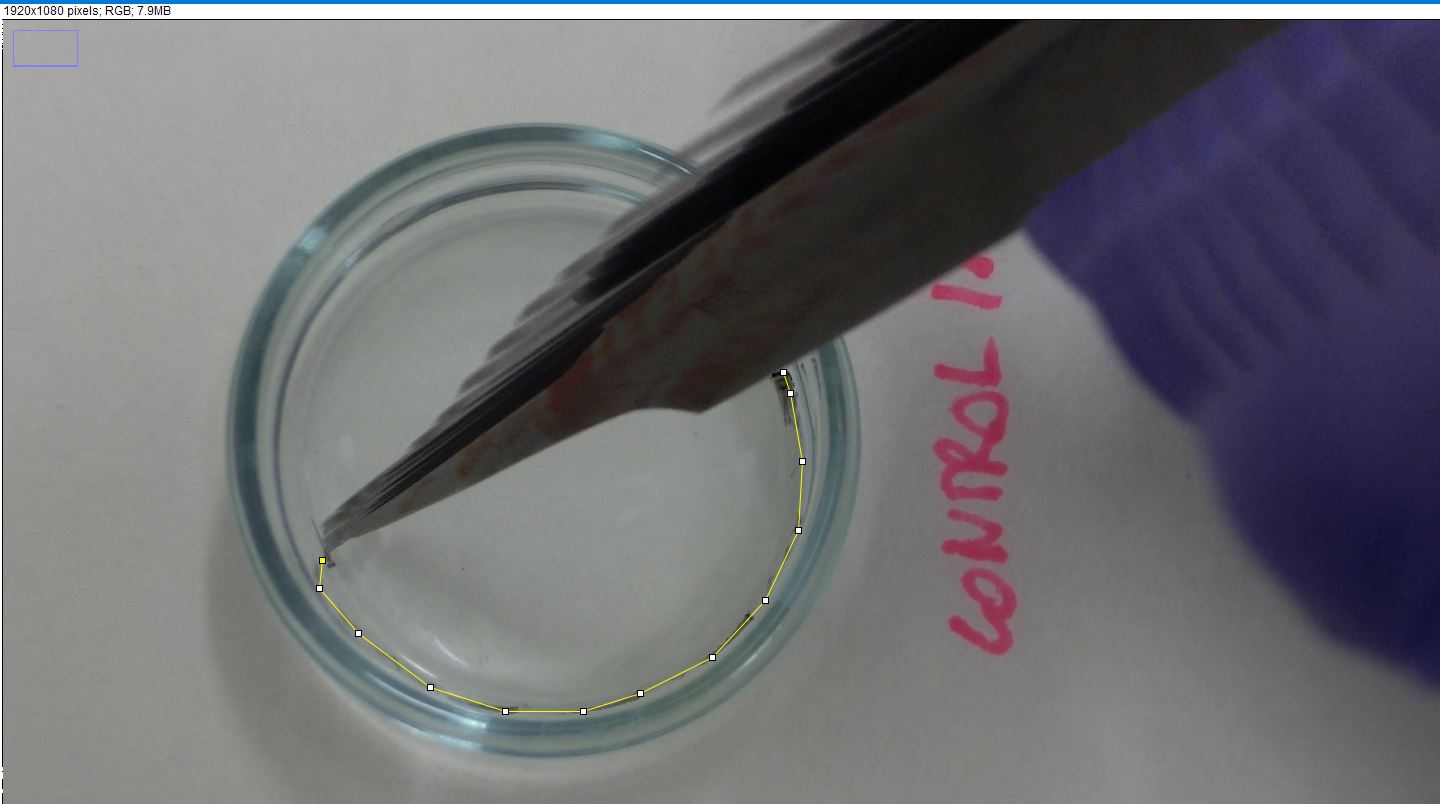

Supplement: Supplementary file 1 [file pharmaceuticals-18-00488-s001.zip › CNT_2_1.JPG]

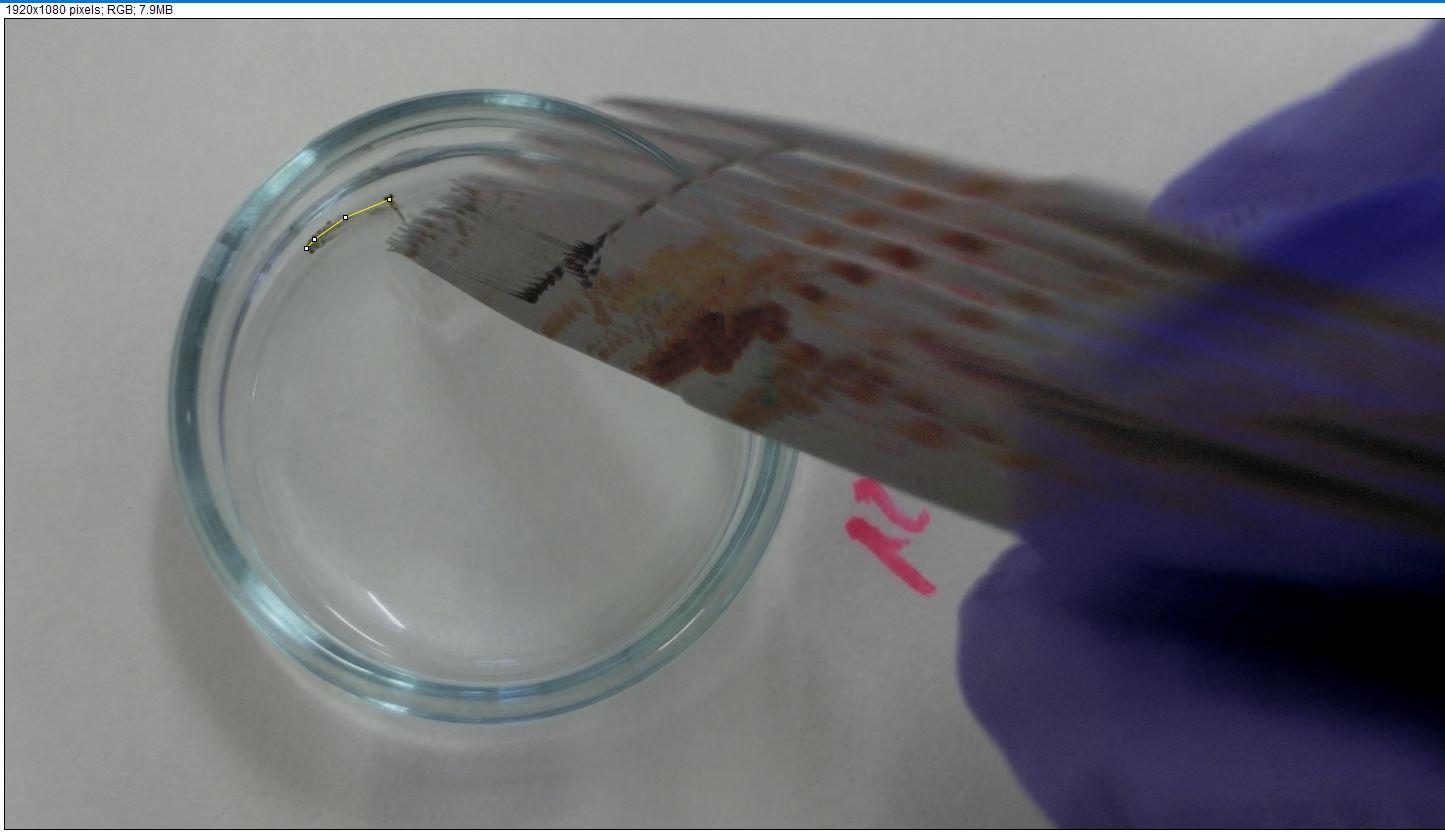

Supplement: Supplementary file 1 [file pharmaceuticals-18-00488-s001.zip › CONC12.5_1_3.JPG]
